# Beyond Grades: Temperament and Interests, but Not School Grades, Highlight Distinct Polymathic Learning Abilities

**DOI:** 10.3390/jintelligence14020026

**Published:** 2026-02-05

**Authors:** Irina N. Trofimova, Michael E. Araki

**Affiliations:** 1 Department of Psychiatry and Behavioral Neurosciences, McMaster University, Hamilton, ON L8S 4L8, Canada; 2 School of Management and Governance, UNSW Sydney, Sydney, NSW 2052, Australia

**Keywords:** polymathy, temperament, intelligence, impulsivity, aptitudes and interests, grades, attention regulation, behavioural plasticity

## Abstract

Polymathy relates to the exceptional learning abilities, in which individuals cultivate and coordinate Breadth, Depth, and integrative capability across multiple domains. It builds on mechanisms typically associated with intelligence, including abstraction, problem solving, and the transfer and integration of information. Because polymathic disposition has partial biological underpinnings, it may intersect with other biologically based individual differences, such as temperament. Biographical accounts also indicate that many polymaths did not achieve exceptional school grades, raising questions about whether the multiplicity of interests in polymaths is associated with distractibility and impulsivity, or whether there is a deeper institutional mismatch between polymaths and educational systems. Our study examined these issues using estimated high school grades across three subject areas, documented university grades, a neurochemistry-validated temperament assessment (Structure of Temperament Questionnaire; STQ-77), the Trait Polymathy Scale (TPS), the Barratt Impulsivity Scales (BIS-11), and information about aptitudes and interests from 296 participants (M/F = 152/144). Contrary to speculation that polymathy reflects distractibility, the TPS correlated negatively with the BIS-11 Lack of Attention scale and positively with the STQ-77 scales of Intellectual Endurance and Probabilistic Processing, confirming high sustained attention in polymaths. TPSs also had selective negative correlations with the STQ-77 Neuroticism scale and positive correlations with the STQ-77 Plasticity, Social Endurance, Sensation Seeking, dispositional Satisfaction scales, as well as several specific and general aptitudes and interests. These findings refine the dispositional profile linked to polymathy, highlighting the differential nature of the three components of polymathy.

## 1. Introduction

### 1.1. Biological Basis of Polymathy, Temperament, and Intelligence Suggest a Possible Common Etiology

Polymathy refers to a pattern of intellectual engagement in which individuals develop and apply knowledge across multiple domains (Araki, 2018, 2020; Burke, 2012; M. Root-Bernstein & Root-Bernstein, 2023; R. Root-Bernstein & Root-Bernstein, 2020). Polymathy builds on mechanisms typically associated with intelligence, including high learning abilities, efficient information processing, abstraction, problem solving, and the transfer and integration of information (R. Root-Bernstein, 2003). Moreover, there is a possible factor of emotional and motivational regulation. In the first treatise on polymathy in 1603, it was described as a sustainable self-motivation and self-directed learning (Araki, 2025). Historical figures such as Leonardo da Vinci, Karl Gauss, and Michael Lomonosov illustrate this pattern through their sustained engagement with diverse fields and their substantial contributions to both scientific and artistic domains (Burke, 2020).

The triadic approach to polymathy (Araki, 2015, 2018, 2025; Araki & Cotellessa, 2020) identifies three conjunctive dimensions—Breadth, Depth, and Integration—as the defining features of polymathic development. Breadth is the horizontal dimension of polymathy, reflecting the scope of fields and experiences an individual engages with. It involves not only disciplinary variety but also diversity of contexts: vocational, avocational, interpersonal, and experimental (R. Root-Bernstein & Root-Bernstein, 2020). Depth is the vertical dimension of polymathy, denoting serious engagement within a domain that leads to expertise and potential mastery. It contrasts with superficial dabbling and is central to distinguishing polymathy from dilettantism (R. Root-Bernstein, 2009). Integration is the relational dimension of polymathy, capturing the processes through which heterogeneous knowledge and experiences produce useful synergies (R. Root-Bernstein, 2003). It ranges from discerning underlying patterns and revealing links with previously unconnected elements to bridging communities of practice and translating insights across disciplinary or functional boundaries. Integration is what transforms polymathic Breadth and Depth into generativity: without it, polymathy risks becoming a disjointed accumulation of knowledge (Araki, 2018, 2025; R. Root-Bernstein & Root-Bernstein, 2020). In this study, we used TraitPolymathy Scales (TPSs) to screen for these three polymathy components.

Another expression of polymathy that could be measured is the multiplicity and type of aptitudes and interests of a person. Polymaths can have various interests, not necessarily related to science; however, they are attracted to the areas where integration of knowledge from multiple disciplines is especially salient, such as philosophy. In this study, we used the screening for aptitudes and interests to complement the TPS screening. Polymathic disposition should include biological components, as many documented polymaths exhibited pronounced expansive cognitive tendencies that cannot be fully explained by the socio-cultural conditions in which they developed. Since many biological systems of behavioural regulation are interconnected, it is essential to identify potential overlaps between the intellectual dispositions of polymaths and other biologically based individual differences, such as temperament.

Temperament represents the most reliable predictors of consistent behavioural patterns (Strelau, 1998; Kagan, 2018; V. M. Rusalov, 1989). In contrast to personality, temperament differences are present not only in adult, acculturated humans but also in pre-cultural individuals (animals, infants) (V. M. Rusalov, 1989; Strelau, 1998; Trofimova, 2016), i.e., they exist before socio-cultural factors start regulating behaviour. In other words, similar to polymathic tendencies, temperament profiles also emerge early in life and, in healthy development, stay consistent in relation to individuals’ peers of the same age.

The aim of our study was to investigate the possible associations between polymathy and school grades, aptitudes and interests, twelve temperament traits, and several types of impulsivity. For our study, we used the Structure of Temperament Questionnaire (STQ-77), developed initially from psychophysiological studies of adult temperament (V. M. Rusalov, 1989; V. Rusalov, 2018; V. M. Rusalov & Trofimova, 2007/2023; Trofimova & Sulis, 2011) and upgraded using the neurochemical framework “Functional Ensemble of Temperament” (FET). The FET highlights the high complexity of neurochemical biomarkers of consistent individual differences and the limitations of linear statistical models to represent this complexity (Netter, 2021; Sulis, 2018; Trofimova et al., 2018, 2022). The FET summarizes the teaming of most consistent findings in functional neurochemistry related to the interactions between neurotransmitters, opioid receptors, and hormones in behavioural regulation of specific aspects of behaviour. The FET, therefore,, provides the content validity for the STQ-77 components (Trofimova, 2016, 2018, 2019, 2021, 2022; Trofimova & Robbins, 2016; Trofimova & Gaykalova, 2021).

The 3 × 4 grouping of the 12 FET and STQ-77 components corresponds to three major brain regulatory systems (orientation, speed of integration, and energetic maintenance), each having a separation into four additional components related to mental (probabilistic, related to uncertainty, complexity, and novelty), social–verbal, physical–motor, and emotional aspects of behaviour. The FET and STQ-77, therefore separated probabilistic aspects as Probabilistic Processing, Plasticity and Mental–Intellectual Endurance (sustained attention); social aspects as Empathy (a form of social behavioural orientation), Social–Verbal Tempo and Social–Verbal Endurance; physical aspects as Sensation Seeking, Motor–Physical Tempo and Motor–Physical Endurance; finally emotional aspects are considered in the FET as amplifiers of orienting alertness (Neuroticism), rough integration of actions (Impulsivity) and approval of maintenance of a chosen behaviour (dispositional Satisfaction).

Evidence for construct, concurrent, and discriminatory validity of various language versions of the STQ was demonstrated through significant correlations with internationally known scales (see V. M. Rusalov & Trofimova, 2007/2023; STQ Website, 2007 for reviews). Estimated high school grades, speed of reading and writing, time of performance on the verbal classification tasks, clinical and neurochemical investigations. The STQ-77 has been utilized in a variety of contexts, such as (i) organizational settings (selection of candidates for a job or allocation of the staff; stress conditions at work, uncertainty conditions at work, and management style), (ii) educational, and (iii) clinical settings (V. M. Rusalov & Trofimova, 2007/2023).

For this study, we used the Portuguese version of the STQ-77 (STQ-77Pt), which was developed in 2018–2019 in two series of forward- and back-translation using two Brazilian samples. The factor structure of the Portuguese version of STQ-77 (STQ-77Pt) was investigated earlier and was consistent with the English and Russian versions of the STQ-77Pt (Araki & Trofimova, 2021). Our previous studies investigated the concurrent validity of the orientation scales of the STQ-7Pt: Sensation Seeking scale using Zuckerman Sensation Seeking Scales; Empathy scale using the Questionnaire of Cognitive and Affective Empathy; and Probabilistic Processing using Locus of Control Scales, as well as Schutte’s Emotional Intelligence Scale and Interests in Games (Trofimova & Araki, 2024). The STQ-77Pt scales related to the speed of Integration (Plasticity and Tempo) and endurance (Physical, Social, and Intellectual Endurance) were investigated using the Strelau Pavlovian Temperament Survey, Trait Polymathy Scales, and Eysenck Personality Inventory (Trofimova & Araki, 2022, 2024), all demonstrating good concurrent validity of the studied STQ-77Pt scales.

### 1.2. Polymathy and Probabilistic Processing Aspects (Intelligence)

Three probabilistic FET components were distinguished based on the evidence of the differences in their neurochemical and neuroanatomic biomarkers (Trofimova, 2016, 2019; Trofimova & Robbins, 2016). The FET describes Probabilistic Processing as regulated by cortical–hippocampal glutaminergic, cholinergic, and noradrenalinergic systems supervising other neurotransmitters; Behavioural Plasticity, as regulated by cortico-striatal dopaminergic and cholinergic systems, with support of gamma-aminobutyric acid (GABA) and serotonergic systems; sustained attention (Intellectual Endurance), as supervised by cortical and basal forebrain cholinergic, noradrenalinergic, and serotonergic systems (Trofimova, 2016, 2019, 2021, 2022; Trofimova & Robbins, 2016). It is natural to expect that these probabilistic FET components can contribute to polymath’s intelligence, aptitudes, and interests related to the most complex aspects of knowledge, such as philosophy, mathematics, and science. These interests and aptitudes can influence success during schooling. The most common findings in studies of temperament-grades associations are: sustained attention (known as temperament traits of Persistence (Martin & Holbrook, 1985), Rusalov’s concept of Intellectual Ergonicity (V. M. Rusalov & Trofimova, 2007/2023), Rothbart’s concept of Effortful Control, or temperament trait of Mental Endurance (Trofimova & Sulis, 2011; Trofimova et al., 2026), as well as impulse control and intelligence, which are associated with higher grades.

A: *Probabilistic aspects hypothesis*: It is expected that the three FET componentsrelated to frontal–cortical systems (Probabilistic Processing, Behavioural Plasticity, and Intellectual Endurance), as well as Sensation Seeking, will show significant positive correlations with general (multiple) aptitudes and general (multiple) interests.

### 1.3. Polymathy and School Grades

Given that polymathy draws high learning capacities commonly associated with intelligence, one might expect individuals with a high polymathic disposition to exhibit high school grades. However, such expectations can be confronted with the literature indicating that intelligence is not always associated with high grades (Barbier et al., 2019; Reis & McCoach, 2000; Sternberg & Grigorenko, 2003). Moreover, school and even college teachers are not trained testers. There is no system of quality control preventing subjective biases. There is also a possible ceiling effect in grades, which can equate good performance with exceptionally good performance due to the upper cutoff in grading scales (for example, in a regular art class, both the Mona Lisa and a technically competent figure-by-numbers could receive an A).

Biographical recollections of polymaths suggest that they were often not exceptional students, even though their learning abilities were recognized early in their lives. They were often penalized for not being sufficiently focused on one subject but paying attention to multiple subjects they came across (Araki, 2018; Cotellessa, 2018). This could suggest that the breadth of interests in polymathic individuals could be associated with high distractibility and poor impulse control. An alternative view suggests that learning in multiple disciplines requires reasonable, sustained attention and good impulse control. It is unclear, therefore, to what degree the multiplicity of interests in polymaths is associated with distractibility, impulsivity, sustained attention, and their schooling grades.

B: *Polymathy-schooling hypothesis*: The associations of polymathy with estimated high-school grades (EHGs) and performance on one of the University courses might be the most transparent in school subjects related to science and mathematics.

### 1.4. Polymathy and Emotionality

Moreover, the association between intelligence and emotionality (i.e., a core aspect of personality and temperament) appears complex and potentially contingent on other factors. Some studies report lower neuroticism among higher-intelligence individuals (Bartels et al., 2012). Other work, however, links high intelligence to elevated rates of affective disorders, anxiety, and physiological sensitivity (Karpinski et al., 2018), and still others suggest a heterogeneous pattern in which high intelligence is observed alongside both high and low anxiety (Coplan et al., 2012). These emotionality differences are consequential because negative emotionality is commonly associated with lower academic grades (Li et al., 2009; Fernández-Vilar & Carranza, 2013; Martin & Holbrook, 1985; Nasvytienė & Lazdauskas, 2021). Interestingly, when compared to intelligence, temperament traits (named as “activity,” “adaptability,” “approach-withdrawal,” “distractibility,” and “persistence”) appeared to be a stronger factor in children’s achievements than their intelligence (Martin & Holbrook, 1985). The associations between polymathy and impulsivity, sustained attention, and school grades also need clarification to address the noted controversy. To date, no study has investigated associations between emotionality-related temperament traits and polymathy.

C: *Emotionality aspects hypothesis*: In relation to impulsivity, it is expected that polymathy has negative associations with the Barratt Impulsiveness Scale (BIS-11) and Structure of Temperament Questionnaire (STQ-77) Impulsivity scales. The rationale is that learning the knowledge in Depth (as in polymathy)—in contrast to just having a glimpse of it while being distracted by something novel (as in impulsivity)—requires strong impulse control. Consistent with this logic, we further expect significant negative associations of Impulsivity-related scales with Intellectual Endurance and Probabilistic Processing. Conversely, impulsivity is expected to be positively associated with Sensation Seeking, consistent with prior work linking impulsive tendencies to the pursuit of stimulation and novelty (Zuckerman, 2014). Finally, with respect to emotional valence, we expect polymathy to be positively associated with emotional security, reflected in lower Neuroticism and higher dispositional Satisfaction.

### 1.5. Activity-Specific and Component-Specific Associations

This section advances two complementary sets of predictions. First, we propose that achievement and engagement across different types of activities should align with distinct temperament traits. Second, we derive component-specific expectations for how Breadth and Depth relate to key regulatory tendencies within the Functional Ensemble of Temperament (FET) framework.

D: *Activity-specific hypothesis*: This hypothesis proposes an activity-specific pattern of associations across estimated school grades (EHGs), documented university grades (DU), and temperament traits. Specifically,

- The EHG and DU grades in science–mathematics-related assignments and similar interests and aptitudes with the temperament scales of Intellectual Endurance, Plasticity, and Probabilistic Processing.
- The EHG and DU grades in social activities, social interests, and aptitudes with the temperament scales of Social–Verbal Endurance and Social–Verbal Tempo.
- The EHGs in athletic activities and similar interests and aptitudes are associated with the temperament scales of Motor (physical) Endurance and Motor Tempo.

E: *Polymathy components hypothesis*: Within the Triadic Approach to Polymathy (Araki, 2025), Breadth, Depth, and Integration should impose different regulatory demands on cognition, emotionality, and behaviour. Guided by the FET framework, we expect differentiated associations with Behavioural Plasticity and Sensation Seeking. Specifically, breadth of knowledge should be positively associated with Behavioural Plasticity and Sensation Seeking, insofar as it involves seeking novelty in both knowledge and experiences. By comparison, the Depth dimension of polymathy should be associated with sustained attention and mental endurance, reflecting the capacity to maintain prolonged engagement with a meaningful activity.

## 2. Materials and Methods

Sample: a total of 296 participants (M/F = 152/144, Mean age = 30.71, SD = 11.74), including: 105 undergraduate students enrolled at the entrepreneurship programme in Universidade Federal Fluminense, Brazil, who received course credit in a general management course; 191 professionals (managers or technicians) who volunteered for this study. All subjects received a debriefing and signed an informed consent form before testing. University students received practicum credits for their participation. Statistical processing included calculations of the descriptive scale statistics (means, SD, and confidence intervals), reliability coefficients (Cronbach’s alphas), and correlations among all the measures applied. This study was approved by the Ethics Committee of the author’s university.

### Measures

*Trait Polymathy Scale (TPS-S), Portuguese* (Trofimova & Araki, 2022). The TPS-S is a shortened version of TPS. It measures polymathic tendencies and has a total of 9 statements assigned to three subscales with three items each: Depth of learning (P-D); Breadth of learning (P-B); and Integration (P-I). The index “Total Trait Polymathy” (TPS) sums the scores of all three dimensions. Responses are recorded in the same Likert scale format as in the STQ-77. In this study, the TPS descriptive statistics was for P-D: M = 15.56, SD = 3.16, α = 0.66; for P-B: M = 17.23, SD = 2.80, α = 0.72; for P-I: M = 16.63, SD = 2.59, α = 0.68, and for TPS: M = 16.47, SD = 1.86, α = 0.74.

*Compact Structure of Temperament Questionnaire, Portuguese (STQ-77Pt)* (V. M. Rusalov & Trofimova, 2007/2023; Araki & Trofimova, 2021; STQ Website, 2007) has 77 statements, assigned to 12 temperamental scales (6 items each), and the validity (social desirability) scale (5 items). Responses were recorded in a Likert scale format: “strongly disagree (1),”“disagree (2),”“agree (3),”“strongly agree (4)”. The STQ-77 scales are grouped in line with the neurochemical framework FET as follows:

The *probabilistic group* of scales is related to behavioural regulation in complex and/or novel situations: Intellectual (Mental) Endurance (ERI), known as sustained attention (reliability index Cronbach’s alpha (α) for this scale in this study was 0.73); and Plasticity (PL, α = 0.70) and Sensitivity to Probabilities Scale (PRO, α = 0.70), oriented towards gathering and analyzing information about frequency and significance of events in order to plan future activities.

The *social–verbal group* of scales related to social–verbal aspects of behaviour: Social–Verbal Endurance (ERS, α = 0.74), known as sociability; Social–Verbal Tempo (TMS, α = 0.74), as an ease of speech production; and Empathy (EMP, α = 0.71), as a behavioural orientation on other people’s needs and emotions.

The *physical–motor group* of scales related to physical–motor aspects of behaviour: Physical–Motor Endurance (ERM, α = 0.76); Physical–Motor–Tempo (TMM, α = 0.78), referring to how quickly a person can integrate motor actions; and SensationSeeking (SS, α = 0.79), assessing the extent to which individuals seek physical pleasure, risks and sensational experience to induce an HPA axis arousal.

The *emotionality group* of scales: dispositional Satisfaction (SF, α = 0.71) as an over-optimistic view on own abilities and outcomes of events and over-confident judgments; the Impulsivity Scale (IMP; inability to control immediate impulses for action; α = 0.76); and the Neuroticism Scale (NEU, α = 0.73) assessing dispositional bias in expectations of adverse outcomes of events. None of these scales alone measures anger, depression, or aggression because these three emotions are composed of several components, including low SF and low levels of some of the functional aspects (Endurance, Plasticity) of behavioural regulation (V. M. Rusalov & Trofimova, 2007/2023).

The *social desirability scale*—also used as a validity scale—assesses the degree to which an individual prefers to over-report his/her following social norms. Scores within the range 15–20 are considered invalid due to a tendency to make a good impression by providing biased answers.

*Estimation of High-school Grades(EHG):* participants were asked to estimate their high school grades in three types of subjects using a scale from 1 (lowest) to 4 (highest). The score estimates were recorded for athletics (EHG-At), socio-verbal assignments (EHG-Soc), and mathematics- and science-related subjects (EHG-Sci). This method has been employed in other studies (Trofimova & Sulis, 2011) and is particularly useful in the analysis of intelligence. This method asks for recollection of facts related to documented and potentially verifiable assessment of people’s performance by institutions, and not their subjective thoughts, feelings, or experiences related to this performance. Schooling is one of the most important functional activities of children and young adults. Schooling grades can be considered as natural experimental evidence of individual differences in performance on specific tasks. In this sense, EHG is not a self-report and can have a reasonable validity as a behavioural method in investigations.

*Documented university (DU) grades:* Four grading components were assessed. First, exam grades (Exam) refer to written exams administered during the regular class period. These exams included multiple-choice questions as well as open-ended questions about the class material. The second component referred to Class Participation, Participation, and Positive Involvement (Participation). Although this grade was intended to assess the quality and consistency of the students’ contributions to the class throughout the semester, the most objective component of the grade was the students’ Participation, which carried significant weight in this grade component. Third, in one of the semesters, students did not have assignments. Instead, they had to research, prepare, and deliver two oral presentations about a topic of their choice. Students were graded regarding the quality and depth of their material, their organization, and their presentation skills. The final grade (GrFinal) was a weighted score of the grading components, including performance on all class assignments, i.e., tasks relevant to the course content, whereby students had the opportunity to substantiate their learning. Assignments primarily involved essays, self-reflections, and reports on a relevant aspect of the course content.

*Aptitudes and interests*: These were measured using a slider-based instrument in which participants provided estimates of their aptitudes and interests for the following domains: (i) business, (ii) arts, (iii) technology, (iv) mathematics, (v) social sciences, (vi) life sciences, (vii) sports, (viii) philosophy, (ix) law, and (x) religion. Each domain was represented by two items, one assessing perceived aptitude (self-evaluated capability or potential proficiency) and one assessing a participant’s interest (attraction or motivational engagement), with responses recorded on a 1–10 slider that required participants to position a marker along ten discrete points. Items were presented in randomized order to minimize order effects, and all responses were stored as numeric values representing each participant’s domain-specific self-appraisals. Averages for each category were recorded as general_aptitude and general_interests variables. Two indices were calculated: (I) humanities, averaging scores in the arts, social sciences, law, and philosophy domains, and (II) exact sciences, covering technology, life sciences, and mathematics. Finally, a 4-point Likert scale asked whether participants considered themselves a “person of interests in multiple domains” as an alternative measure of polymathy.

*Barratt Impulsiveness Scale (BIS-11), Portuguese version* (von Diemen et al., 2006): The BIS-11 provides a total score of impulsivity and has three sub-scales: Lack of Planning (LoP, ten items); Lack of Attention (LoA, six items); and Motor Impulsivity (MoM, seven items). Responses were recorded in the same Likert scale format as in the STQ-77. Only protocols from 153 participants (M/F = 95/58, Mean age = 25.37, SD = 9.28) were recovered.

*Statistical analysis* included descriptive statistics (means, SD), reliability coefficients (Cronbach’s alphas) for all used scales and analyses. The study is adequately powered to detect small-to-moderate associations in the range typically reported in individual-differences research. For a bivariate association of *r* = 0.25, our a priori power calculation indicates that 124 observations are sufficient for 80% power at α = 0.05. While concerns about sample heterogeneity exist, they primarily affect the precision of subgroup comparisons and interactions, which are not the primary focus of the present analyses. The main effects reported here are estimated in the pooled sample, and key sources of confounding are limited by design: education is already controlled for in the undergraduate sample, and the professional sample exhibits relatively little educational variation, consisting largely of degree-holding senior professionals.

For Statistical analysis, we used Statistica 10 and Excel software.

## 3. Results

Table 1 shows the descriptive statistics for the STQ-77Pt and BIS-11 and their correlations with the TPSs and the estimated high-school grades (abbreviated earlier as EHG). Table 2 presents the correlations between the TPS scales and EHG, and documented university grades (DU), as well as aptitudes and interests. The means (M) and standard deviations (SD) of the grades were for science–mathematics: M = 2.93, SD = 0.83, Social–Verbal assignments: M = 3.06, SD = 0.86, Athletics: M = 3.09, SD = 0.95.

All TPSs showed statistically significant positive correlations with the STQ-77 scales of Intellectual Endurance, Plasticity, and Probabilistic Processing, and negative correlations with the Lack of Attention scale of the BIS-11. The scales of Breadth, Integration, and Total of TraitPolymathy Scales showed significant positive correlations with Sensation Seeking, Social Endurance, Social Tempo, and dispositional Satisfaction, and negative correlations with the Neuroticism scales of the STQ-77.

No significant correlations were found between the TPS and EHG in this study. However, there was an activity-specific differentiation in correlations between the EHG and the temperament scales. The EHGs in science and mathematics showed high positive correlations with the STQ-77Pt scales of Probabilistic Processing and Intellectual Endurance, and a negative correlation with the STQ-77 Neuroticism and Lack of Attention scale of the BIS-11. The EHG in social subjects correlated positively with Social–Verbal Tempo and Empathy scales of the STQ-77. The EHG in physical–athletics subjects had significant positive correlations with the STQ-77 scales of Motor Endurance and Motor Tempo, and negative correlations with Intellectual Endurance. A similar activity-specific pattern was observed in the associations between the EHG and aptitudes and interests. The EHG in science and math subjects had positive associations with technical and mathematics aptitudes/interests and exact sciences interests, and a negative correlation with interests in humanities. Estimated high school grades in physical/athletic subjects correlated positively with sports aptitudes and interests, and multiplicity of interests. The EHG in Social activities/subjects had a significant positive correlation only with life sciences aptitude (r = 0.24, *p* < 0.05) (Table 2).

Similarly, in the DU—STQ-77 correlations (Table 3), Intellectual Endurance and Probabilistic Processing showed positive correlations with Assignment, Oral Presentation, and Grade Average. Additionally, Probabilistic Processing had positive correlations with Exam and Participation in documented adult performance on a university course. The Social Endurance and Social Tempo scales of the STQ-77 showed a high positive correlation with Oral Presentation. Sensation Seeking had a negative correlation with Assignment and Plasticity, and a negative correlation with Participation on DU.

All polymathy scales had significant positive correlations with “multiplicity of interests”, “mathematics interests”, “philosophy interests”, and summing measures of aptitudes and interests (“general aptitude” and “general interest” variables), indicating the number of an individual’s aptitudes and interests (Table 2). Both aptitudes and interests in philosophy and law had positive associations with the Integration and Total scales of the TPS. The Integration–polymathy scale of TPS also had significant positive correlations with arts aptitudes/interests, life sciences aptitudes, religion aptitudes/interests, as well as interests in the humanities and social sciences. Technical aptitudes had a positive correlation with Breadth–polymathy, and social sciences aptitudes with Depth–polymathy scales. Business aptitudes and interests, and life science interests did not have significant correlations with TPS or EHG and were not included in Table 2.

The BIS-11 Lack of Attention scale had a significant negative correlation with the DU Grade Average, social sciences, philosophy, law, and general (total number of) aptitudes and interests, and also with interests in humanities (Table 2). Additional effects (all at *p* < 0.05), included the negative associations between the BIS-11 LoP scale and philosophy interests (*r* = 0.24), BIS-11 MoM (*r* = 0.24), as well as Total scales (*r* = 0.26) and math interests, and positive correlation between the sports interests and MoM of the BIS-11 (*r* = 0.23).

Table 3 shows the correlations of the documented university grades and BIS-11 scales with the STQ-77 scales and the DU-EHG correlations. All four scales of the BIS-11 had negative correlations with Grade Average in documented adult university grades.

The BIS-11 Lack of Planning scale correlated negatively with Assignment grades, the Lack of Attention scale correlated negatively with estimated high-school grades in science and mathematics, and the Total BIS-11 scale correlated negatively with performance on an Exam. Grade Average in DU also had a positive correlation with the EHG in science and mathematics.

Two probabilistic scales of the STQ—Intellectual Endurance and Probabilistic Processing—was high negative, and Impulsivity had high positive correlations with the BIS-11 LoA, MoM, and BIS-11 Total scales. Sensation Seeking had a high positive correlation with the LoP, MoM, and BIS-11 Total scales, Social Endurance—with MoM and BIS-11 Total scales, Empathy—with LoP and BIS-11 scales, Satisfaction—with Lack of Planning, and Neuroticism—with Lack of Attention and Motor Impulsivity scales of the BIS-11. BIS-11 Lack of Planning, however, correlated negatively with STQ-77 Neuroticism. Other grade-related variables did not have significant correlations with BIS-11.

There are multiple significant correlations between the STQ-77 scales and the measured aptitudes and interests (Table 4) that can be grouped using an activity-specific approach, separating them into intellectual, social, physical, and emotional aspects.

Intellectual Endurance and Probabilistic Processing showed high positive correlations with general scores of aptitudes and interests, as well as the multiplicity of interests, interests in mathematics, social sciences, philosophy, and law. Intellectual Endurance also showed positive correlations with aptitudes and interests in mathematics and the social sciences, as well as interests in the humanities. The Probabilistic Processing scale had an association with tech aptitudes and interests. In contrast, the Plasticity scale correlated positively with interests in humanity, religion, and the general index of interests.

Social–Verbal Endurance had positive correlations with art aptitudes/interests and interests in religion, whereas Social–Verbal Tempo did not show any significant correlations with aptitudes and interests at all. Empathy scale, however, had a negative correlation with business aptitudes and interests and a positive correlation with interests in social sciences, sports, and philosophy.

Motor–Physical Endurance, Motor Tempo, Sensation Seeking, and Impulsivity scales of the STQ-77 had significant positive correlations with sports aptitudes/interests. Motor-physical Endurance and Motor Tempo had positive correlations with business aptitudes, and Motor Endurance—also with multiplicity of interests and tech interests. Sensation Seeking also had a positive correlation with arts aptitudes.

The scale of Satisfaction related to emotional valence did not have any significant correlations with estimations of aptitudes and interests; however, the STQ-77 Neuroticism scale had negative correlations with interests in mathematics and both aptitudes and interests in philosophy, religion, and general indices of aptitudes and interests.

## 4. Discussion

This study examined the associations between polymathy and its expression in academic performance, reported aptitudes and interests, and biologically based traits as assessed by a neuroscience-based test. Table 5 summarizes the differential association of polymathy components with temperament traits, the BIS-11 Lack of Attention scale, aptitudes, and interests.

*Our A (Probabilistic) hypothesis* was supported as the results showed the proposed associations between scales of TraitPolymathy Scales and the STQ-77 scales of Intellectual Endurance, Probabilistic Processing, Plasticity, and Sensation Seeking. Moreover, the positive correlations of all polymathy scales with multiple mathematics and philosophy interests, as well as summing measures of aptitudes and interests (general aptitudes/interests support the idea that polymaths have an orientation to learn systematic and complex laws of nature and society.

This finding is consistent with the view that philosophical aptitudes and interests reflect a drive toward multidimensional analysis of available information. Interest in philosophy is unlikely to be sustained in the absence of the cognitive capacities required for it, particularly probabilistic processing and sustained attention, which seem to be a signature of individuals high in polymathic disposition.

Remarkably, there were no correlations between either estimated high-school grades or documented university grades and any of the polymathy scales. In other words, *our hypothesis B*, which suggested that polymaths may achieve higher science and math-related grades compared to others, was not supported. This is consistent with biographical observations that the success of polymaths in science and society does not correlate with their achievements in school (Cotellessa, 2018; Araki, 2020, 2025). Polymaths were often not the best students due to their restless yet defiant curiosity, tendency to question authority, and inclination to explore beyond prescribed curricula. There could be at least three explanations forour results and biographic observations:

(1) Our methods were flawed, and similar patterns in biographic observations were just a coincidence. In this case, more studies are needed to verify this result.
(2) There are potential mismatches between polymathic abilities and conventional educational structures. Conventional educational and occupational systems are designed to cultivate, structure, withthe tendency of incentives and evaluation criteria to privilege curricular compliance, short-horizon performance, and strategic optimization of evaluation rules.Polymathic individuals, however, yearn for expansive exploration coupled with deep dives and integrative development regardless of domains. They may appear “underrecognized” in grade-based systems, not because they lack capacity, but because their learning and achievement are expressed through patterns—range, synthesis, and long-horizon competence building—that grades were never designed to measure. Rather than excelling through compliance with standardized education, many polymaths are autodidacts, showing uneven educational records, with underperformance in formal schooling often offset by extraordinary self-directed learning and (later) creative accomplishments.
(3) Associations between the PRO scale and the Polymathy scales (and emotional security, as discussed below) suggest that even though polymaths had high learning abilities, they preferred to adopt a “good enough” approach when it came to school grades. In short, polymathic knowledge is much more than school grades.

In this sense, achievements as measured by the school or university grades might not be good screening parameters when we are looking for polymaths.

*Our Emotionality-related hypothesis C* included two parts: impulsivity-related and emotional valence. Both parts of this hypothesis were supported by our results, suggesting good impulse control in polymaths(opposing the idea that polymaths are “easily distracted” by various sources of information and so only look knowledgeable in this information). In terms of impulsivity, all polymathy scales had statistically significant negative correlations with the Lack of Attention scale of the Barratt Impulsivity Scales, which, in turn had negative correlation with the documented Grade Average in adult learners and negative associations with aptitudes and interests in social sciences, philosophy, math, law and general indices, i.e., with all variables that correlated positively with the polymathy. Moreover, the BIS-11 Lack of Planning scale correlated negatively with assignment grades; Lack of Attention correlated with estimated high-school grades in science and mathematics; and the Total BIS-11 scale correlated with performance on an exam, confirming the commonly reported association between high performance in school and sustained attention. The TPS—BIS-11 correlations are in line with the TPS correlations with the STQ-77 scale of Intellectual Endurance that measures sustained attention. Therefore, the received pattern of multiple correlation suggests strong sustained attention in polymaths; however, the matter of impulsivity might not be completely sorted. After all, in terms of neuroanatomy, impulse control was linked to the same brain area as sustained attention (the frontal lobes), but, as the FET highlights, these two characteristics have different neurochemistry. Impulsivity depends on the activity of delta-opioid receptors, which activate dopaminergic systems, and is intertwined with the hormonal regulation of behaviour. In contrast, sustained attention, as well-documented, involves cholinergic systems of the basal forebrain and frontal lobes, utilizing GABA and other neurotransmitters as mediators (Trofimova, 2019, 2021).

Moreover, as expected in hypothesis C, there was a significant positive correlation between the BIS-11 Impulsivity scales and the STQ-77 Sensation Seeking scale, in line with the research by Zuckerman (2014), who introduced the concept of sensation seeking to psychology.

*The hypothesis C in relation to emotional valence* was also supported, as the results showed high emotional security associated with polymathy. This association was observed in negative correlations between three TPSs and Neuroticism, and positive correlations with high dispositional Satisfaction scales of the STQ-77. These associations between polymathy and emotionality scales align with the predictions of the neurochemical FET framework on which the STQ-77 is based (Trofimova, 2018, 2021). The basis of this dispositional positive emotionality in polymathic individuals is explained by the FET as an association of integrational aspects of behaviour (high in polymathic individuals) with dopamine release that is activated by mu- and delta-opioid receptors, which, in turn, are linked to positive emotionality and a sense of security (Trofimova, 2018, 2021; Trofimova & Gaykalova, 2021). Our results are also consistent with reported self-confidence in polymathic individuals (Cotellessa, 2018), which likely enables them to venture into unfamiliar disciplines and acquire new knowledge without feeling intimidated (Cotellessa, 2018; Araki & Cotellessa, 2020).

*The activity-specific hypothesis D* was supported in all its components, as there was an activity-specific differentiation in correlations between the EHG, temperament scales, and aptitudes/interests. The activity-specific approach was developed by V. M. Rusalov (1989), V. Rusalov (2018) based on his psychophysiological studies of temperament and subsequently supported by the FET summary of neuroanatomic and neurochemical partitioning of hypothalamic-pituitary and cortical systems (Trofimova, 2021). This approach points to the benefits of separating regulatory systems into “intellectual”, probabilistic (related to not immediately present or complex or novel events), “social” (body-to-body), and physical–motor aspects of behaviour. Our results demonstrated the validity of this approach, as evidenced by the pattern of correlations. As hypothesis D expected, the EHGs in science and mathematics had high positive correlations with the STQ-77Pt scales of Probabilistic Processing and Intellectual Endurance scales, which in turn, correlated positively with the correlates of polymathy, such as general scores of aptitudes and interests, multiplicity of interests, interests in mathematics, social sciences, philosophy, and law. The EHG in science/math subjects also had positive associations with technical and mathematics aptitudes/interests and exact sciences interests, and a negative correlation with interests in humanities. Documented adult university grades on Assignment, Oral Presentation, and Grade Average correlated positively with Intellectual Endurance and Probabilistic Processing, and Probabilistic Processing also had positive correlations with Exam and Participation.

In contrast to this “intellectual cluster”, there were “social” and “physical” clusters of correlations. Social–Verbal Endurance had positive correlations with art aptitudes and interests, as well as interests in religion. The EHG in social subjects correlated positively with the Social–Verbal Tempo and Empathy scales of the STQ-77. Finally, the physical aspects cluster was formed when the Motor–Physical Endurance, Motor Tempo, Sensation Seeking, and Impulsivity scales of the STQ-77 showed significant positive correlations with sports aptitude and interests. The EHG in physical–athletics subjects showed significant positive correlations with Motor Endurance and Motor Tempo, and negative correlations with Intellectual Endurance. A similar activity-specific pattern was observed in the associations between the EHG and aptitudes and interests. Estimated high school grades in physical/athletic subjects correlated positively with sports aptitudes and interests, and multiplicity of interests. Moreover, the Social Endurance and Social Tempo scales of the STQ-77 had a high positive correlation with Oral Presentation.

Our results showed that polymathy, to a large extent, correlates with the “intellectual cluster”, but it would be a mistake to treat polymaths as introverts. There were significant positive correlations between the Depth–polymathy scale and aptitudes in the social sciences, as well as between the Integration–polymathy scale and interests in the humanities and social sciences. The Breadth and Integration scales of the TPS also showed significant positive correlations with the Social–Verbal Endurance and Social–Verbal Tempo scales of the STQ-77. This is consistent with biographical observations of polymaths, who can efficiently use verbal means for their analytic explorations, develop new concepts, and easily communicate with peers when needed. The pattern of correlations between applied measures showed that it makes sense to differentiate between intellectual and verbal abilities—a differentiation that was missing in early tests of intelligence, which provided IQ scores based on people’s verbal fluency and knowledge of specific names and English words. However, people can have both types of abilities (i.e., probabilistic processing and verbal) highly developed, and this might be a case for polymathy.

In line with *our E-hypothesis regarding the different nature of polymathy* components, the TPS Breadth (and not Depth) of knowledge scale had a high positive correlation with the STQ-77 Plasticity and Sensation Seeking scales and technical aptitudes. In contrast, Depth of knowledge had a stronger positive association with interests in math, social sciences, and aptitudes/interests in law, and a negative correlation with the BIS-11 total scale. It can be viewed as breadth of knowledge, i.e., consistent learning of a wide range of disciplines in polymaths can be explained by high Behavioural Plasticity and Sensation Seeking. Moreover, it appeared that TPS-Breadth, but not Depth, is associated with significantly higher dispositional Satisfaction and lower Neuroticism (Table 1). After all, expanding knowledge to unknown disciplines requires a high tolerance for uncertainty, i.e., low Neuroticism and higher self-confidence. However, learning a discipline in Depth does not require emotional security. It is possible that Plasticity, Sensation Seeking, and high stress resilience to possible humiliations during learning complex matters enable polymathic individuals to expand incoming information, integrate various actions, tolerate switches between tasks, and complete them. In contrast, the Depth dimension of polymathy requires stronger impulse control (as seen in negative correlations with BIS-11), which enables them to tackle complex matters in mathematics and the social sciences.

This pattern is consistent with previous research on knowledge building (Hirsch, 2003; Royer et al., 1996; Royer & Cunningham, 1981), supporting the view that comprehension and expert performance depend substantially on accumulated domain knowledge and its organization, with Breadth reflecting the accumulation of wide-ranging knowledge that facilitates comprehension and transfer across contexts, whereas Depth reflects concentrated, controlled investment that supports progressive complex reasoning within a domain.

The Integration component of polymathy appears to be associated with aptitudes and interests in philosophy, law, the arts, life sciences, religion, humanities, and social sciences, and also exhibits specific positive correlations with the STQ-77 scales of Social Endurance and Social Tempo. The Integration component of polymathy may be more closely tied to an individual’s verbal abilities in comparison to the Depth and Breadth components.

In other words, we could expect at least three components of polymathy, with variations in different individuals depending on their temperament:

- Breadth of knowledge is notable in polymaths with high Plasticity, Sensation Seeking, and tolerance to adversity;
- Depth of knowledge in individuals with strong impulse control and less Sensation Seeking or Plasticity, but still having high Probabilistic Processing and Intellectual Endurance, common for all polymaths;
- Integration of knowledge in individuals with a drive to tackle the most complex areas of knowledge, especially related to the interactions between social and physical processes.

A few more observations are worth noting:

(1) It seems that dispositional good mood cannot be an indicator of specific aptitudes or interests since the dispositional Satisfaction scale in our study did not have any significant correlations with estimations of aptitudes and interests. The STQ-77 Neuroticism scale, however, had negative correlations with interests in mathematics and both aptitudes and interests in philosophy, religion, and general indices of aptitudes and interests. We do not share the view that Neuroticism is the same as low Satisfaction (a negative emotional valence) and follow the FET view on Neuroticism as dispositional behavioural alertness (Trofimova, 2021; Trofimova & Gaykalova, 2021). The FET highlights the role of kappa-opioid receptors in Neuroticism, which regulate (among other targets) noradrenaline release needed in attention to novelty. This attention is a key in learning complex matters such as philosophy and mathematics, and polymaths likely have an optimal level of activation of this system. Outside of the optimal range, however, both too low and too high activation of dynorphins and noradrenalin can compromise a person’s ability to comprehend new and complex knowledge.
(2) A use of documented academic grades involves multiple technical difficulties, whereas the use of estimated high school grades (EHG, i.e., asking people to estimate their performance on specific subjects using, say, 1 to 4 or other scale) can be easily arranged. This and previous studies (Trofimova & Sulis, 2011; Trofimova et al., 2026) utilized the EHG in conjunction with the STQ-77 Excel files for testing, which are freely available in 24 languages on the developer’s website (STQ Website, 2007). These testing Excel files include an additional line on the testing temperament form, asking people to estimate their high school grades in three areas. Since this information relates to people’s biographic data, it can be considered as experimental variables. The results of this study, similar to previous studies (Trofimova & Sulis, 2011; Trofimova et al., 2026), validated the EHG method, showing the activity-specific patterns of the EHG correlations with the STQ-77 scales (Table 1); EHG-science correlation with the BIS-11 Lack of Attention scale (Table 1), with the DU Exam and Grade Average in documented university grades (Table 2); and a reliable activity-specific pattern of correlations of EHGs with aptitudes and interests (Table 2). These results validated the EHG method as a reliable and easily used source of biographic information about an individual’s achievements in at least three subject areas of high school classes.
(3) Since our study used a Portuguese-speaking sample of Brazil, there is a question about the cross-cultural equivalence of our results. We used the variables that were either proven to be biologically based (temperament and polymathy scales) or were related to the recorded behaviour (grades) and behavioural preferences (interests and aptitudes). The nature of our variables gives hope that, with replication of our study in other cultures, the results would be compatible.

## 5. Future Directions, Recommendations, and Limitations

### 5.1. Implications for Educators and Practitioners

Building on the broader pattern of findings reported above, the null association between polymathy and grades is informative beyond schooling itself. It can be read as an illustration of a broader institutional failure. Grading systems are designed to sort individuals through standardized, rubric-based signals of performance, yet our results suggest that polymathy is not amenable to that kind of metric. In that sense, grades function as a metaphor for how many modern institutions mishandle polymathy.

First, they struggle to identify polymathic potential because they privilege narrow, highly codified indicators of performance while polymathy often expresses itself through long-range self-directed learning, exploratory breadth, and integrative synthesis—qualities that are not easily captured by standardized criteria.

Second, they struggle to nurture polymathic talent because the same systems that fail to detect it frequently penalize its behavioural signatures: curiosity that exceeds the syllabus, boundary-crossing that appears “unfocused,” and integrative thinking that does not map neatly onto disciplinary checklists. The result is a predictable selection dynamic in which polymathic development is beyond overlooked but actively discouraged.

Third, and most consequentially, this misalignment carries an economic and organizational cost. When institutions operate under narrow evaluation regimes, they systematically underinvest in the very people structurally positioned to generate and leverage cross-domain recombinations and integrative solutions—the cognitive profile that can be most helpful under uncertainty, novelty, and discontinuous change because it uniquely enables exploration, recombination, and renewal. In doing so, organizations and society leave value on the table: fewer genuinely good but non-obvious connections are pursued, fewer latent opportunities survive the gatekeeping, and fewer high-quality alternatives are ultimately implemented before the environment moves on. The long-run consequence is institutional brittleness: organizations become specialized at refining or repackaging what they already know but increasingly fragile when novelty arrives, constraints shift, or discontinuities appear—that is part of the cost of evaluation systems that have systematically selected against polymathic capacity.

### 5.2. Future Directions

Future directions of this type of research can investigate the generalizability of our results using samples in other cultures. Free access to the STQ-77 in 24 languages, its low-maintenance testing and processing procedures with available Excel forms, and the ease of applying the EHG method can facilitate cross-cultural studies.

The arbitrariness in concepts related to individual differences could be reduced by anchoring domain selection in a more systematic mapping framework—for example, an “Atlas of Human Interests” that organizes interest domains, specifies their conceptual distances, and supports cross-cultural comparability. This would strengthen construct validity, improve replicability across studies, and, once implemented, could be offered to students as a navigational tool to better understand the landscape of interests, abilities, and skills anywhere in the world where the Atlas has been implemented.

### 5.3. Recommendations

The recommendations, therefore, include the advice to take advantage of the methods used in our study and to search for culture-specific methods in studying polymathy. The FET-based STQ-77 structure has a unique validation by neurochemical biomarkers and potentially can help to crystallize the overlapping concepts of psychology related to intelligence, distractibility, hyperactivity, and polymathy. We recommend using the STQ-77 to facilitate the insights related to the neurochemical systems of intelligence.

Moreover, we recommend further examining the possible dissociation of polymathy from the school grades seen in our results. This dissociation can be explained by the “distractibility” of polymaths, sharing their attention on multiple areas of knowledge, resulting in not perfecting their grades on specific subjects. However, another explanation might be in the flaws of our method to measure polymathy. Further studies are needed to examine this possibility.

### 5.4. Limitations

The limitations of the studies are related to the self-report nature of the STQ-77 and BIS-11. To overcome these limitations, we used the validity scale of the STQ-77, which screens for social desirability in responses. Additionally, the components of the STQ-77 were linked to neurochemical biomarkers, thereby enhancing the diagnostic power of the test scales. Moreover, for the final summary of the results (Table 5), we set conservative criteria for statistical significance as *p* < 0.01.

Another limitation is related to a possible retrospective bias in EHG. However, from our past use of this method (Trofimova & Sulis, 2011; Trofimova et al., 2026), we observed that people normally do not over- or under-estimate the grades related to their past schooling. We can guess that such honesty might stem from the impression that grades can be easily checked if needed, so “there is no point in lying”. Plus, people tend to consider their performance in school as facts of their past and share the information about EHG the same way as other facts of their childhood. In this sense, estimated grades can still have a reasonable validity as a behavioural method in investigations.

## 6. Conclusions

Polymathy, i.e., a distinctive pattern of intellectual development characterized by the capacity to progress substantially and synergistically across multiple disciplines, remains both a source of fascination and an unresolved scientific puzzle (Araki, 2025). Biographies of polymaths often highlight the early onset of their abilities (Burke, 2020), similar to their temperament, but also reveal a lack of correlation between achievements during schooling and their success in multiple disciplines later in life. Moreover, the speculations that polymathy is associated with distractibility and impulsivity (inability to suppress attention to distractions) should be examined.

Our study examined the associations between polymathy and temperament profiles measured by the Structure of Temperament Questionnaire (STQ-77), which is structured in line with functional neurochemistry and follows an activity-specific approach (i.e., separation between intellectual–probabilistic, social–verbal, and physical–motor aspects of behavioural regulation). We also used the Trait Polymathy Scale (TPS), the Barratt Impulsivity Scales, and estimated high school grades in three areas (mathematics–science subjects, social assignments, and athletics), as well as five documented university grades, to examine the associations between polymathy and impulsivity and school grades.

The results could be summarized as follows:

1. Polymathy was associated with high expression of temperament traits of Intellectual and Social–Verbal Endurance, Plasticity, Probabilistic Processing, and Sensation Seeking, as well as higher aptitudes and interests in philosophy, mathematics, law, religion, and the multiplicity of aptitudes and interests.
2. Despite confirmed associations with these measures of intellectual traits, aptitudes, and traits, polymathy was not associated with any range in estimated or documented grades in schooling. It is possible that polymaths, despite their clear intellectual learning abilities, often go “outside of the box” of standard educational settings, expanding the spectrum of their knowledge, but adopting a “good enough” approach when it comes to school grades. In this sense, achievements as measured by the school or university grades might not be good screening parameters when we are looking for polymathic individuals.
3. Contrary to a presentation of polymathy as distractibility, all polymathy subscales had statistically significant negative correlations with the Lack of Attention scale of the Barratt Impulsivity Scales. In combination with positive correlations with Intellectual Endurance, it suggests strong, sustained attention in polymathic individuals.
4. Contrary to a presentation of polymathy as introversion, there were statistically significant positive correlations between polymathy and Social Endurance, Social Tempo temperament scales, aptitudes/interests in social sciences, and interests in the humanities.
5. Polymathy was associated with emotional security, having negative correlations with Neuroticism and positive correlations with dispositional Satisfaction.
6. The pattern of correlations between polymathy, temperament scales, estimated school grades, documented university grades, and aptitudes/interests supported the activity-specific approach, which suggests separating analysis of individual differences into three types of functional aspects: probabilistic–intellectual, social–verbal, and motor–physical.
7. There was a trend for two components of polymathy to have differential associations with the used scales validating the idea of separation of these components in the structure of polymathy: (a) Breadth but not Depth of learning had significant positive correlations with STQ-77 Plasticity, Sensation Seeking, dispositional Satisfaction and low Neuroticism, likely related to exploratory component of polymathy; (b) Depth but not Breadth of learning had stronger positive association with interests in math, social sciences aptitudes and aptitudes/interests in law, and impulse control as measured by the BIS-11; (c) The Integration component was associated with higher scores in Social Endurance, Social Tempo (and so more coupling with verbal abilities than in Breadth and Depth components), aptitudes and interests in complex areas of knowledge, especially related to the interactions between social and physical processes.
8. The pattern of correlations of estimated high-school grades with the scores of STQ-77, BIS-11, aptitudes and interests validated this method as a reliable and easily used source of biographic information about an individual’s achievements in three subject areas of high school classes.

## Figures and Tables

**Table 1:** Correlations of the Trait Polymathy Scales (TPSs) and the estimated high school grades in science and math subjects (GrSci), social activities/subjects (GrSoc), and athletics (GrPhys) (*N* = 296) with the STQ-77 (*N* = 296) and BIS-11 (*N* = 153) scales. Zeros before the decimal point are omitted. *Note*: Structure of Temperament Questionnaire scales: ERM/ERS/ERI—Motor–Physical/Social/Intellectual Endurance; TMM/TMS—Motor–Physical/Social Tempo; SS—Sensation Seeking; EMP—Empathy; PL—Plasticity; PRO—Probabilistic Processing; SF—dispositional Satisfaction; IMP—Impulsivity; NEU—Neuroticism. Barratt Impulsiveness scales, *N* = 153: LoP—Lack of Planning, LoA—Lack of Attention, MoM—Motor Impulsivity, BIS11—Total; Trait Polymathy Scales: P-D—Depth of learning, P-B—Breadth of learning, P-I—Integration, TPS—Total score. * *p* < 0.05, ** *p* < 0.01, *** *p* < 0.001.

Scales:	*Mean*	*SD*	P-D	P-B	P-I	TPS	GrSci	GrSoc	GrPhys
ERI	16.70	3.46	0.29 ***	0.26 ***	0.22 ***	0.35 ***	0.20 **	0.02	−0.15 *
PL	15.23	3.56	0.12 *	0.36 ***	0.29 ***	0.34 ***	0.02	0.05	−0.02
PRO	18.06	3.49	0.28 ***	0.36 ***	0.23 ***	0.39 ***	0.18 **	0.12	−0.12
ERS	17.35	3.80	0.04	0.15 *	0.21 **	0.18 **	−0.04	0.16 *	0.07
TMS	16.52	3.52	0.05	0.14 *	0.13 *	0.14 *	−0.01	0.21 **	0.10
EMP	15.13	3.44	0.05	0.11	0.03	0.08	−0.01	0.15 *	−0.13 *
ERM	15.25	3.79	0.05	0.12	0.09	0.11	−0.02	−0.06	0.30 ***
TMM	15.40	3.78	0.04	0.14 *	0.07	0.11	0.00	−0.05	0.38 ***
SS	16.08	3.93	−0.05	0.25 ***	0.18 **	0.17 **	−0.05	0.01	0.12
SF	14.36	3.85	−0.01	0.18 **	0.18 **	0.15 *	0.05	0.05	0.10
IMP	16.47	3.64	−0.11	0.00	−0.07	−0.08	0.00	0.00	0.09
NEU	15.30	3.61	−0.11	−0.34 ***	−0.35 ***	−0.36 ***	−0.14 *	−0.05	0.04
LoP	20.20	4.27	−0.13	0.16	0.07	0.05	−0.04	0.05	0.03
LoA	13.23	2.63	−0.21 *	−0.23 **	−0.22 *	−0.32 ***	−0.25 **	−0.07	0.10
MoM	15.46	3.51	−0.13	0.03	−0.02	−0.06	−0.13	−0.09	0.00
BIS11	48.90	7.94	−0.19 *	0.02	−0.04	−0.10	−0.16	−0.04	0.05

**Table 2:** Correlations of the Trait Polymathy Scales (TPSs), the estimated high school grades in science/math subjects (GrSci), athletics (GrPhys) (*N* = 296), and the BIS-11 LoA scale (*N* = 153) with documented adult academic performance on Exams, Grade Average (GradAve) (*N* = 87), aptitudes (*N* = 80) and interests (*N* = 79). The EHG in social activities/subjects had a significant positive correlation only with life sciences aptitude (*r* = 0.24, *p* < 0.05). Note: TPSs: P-D—Depth–polymathy, P-B—Breadth–polymathy, P-I—Integration, TPS—Total score. * *p* < 0.05, ** *p* < 0.01, *** *p* < 0.001.

Scales:	P-D	P-B	P-I	TPS	GrSci	GrPhys	LoA
Exam	0.12	0.04	0.07	0.11	0.36 **	−0.11	−0.20
GradAve	0.12	0.12	0.09	0.16	0.29 **	−0.15	−0.23 *
art_aptitude	0.08	0.16	0.29 *	0.22	−0.11	−0.10	0.10
lifescience_aptitude	0.16	0.13	0.25 *	0.22	0.08	−0.03	−0.09
tech_aptitude	0.15	0.26 *	0.11	0.21	0.33 **	0.08	−0.03
math_aptitude	0.10	0.21	0.18	0.20	0.57 ***	0.06	−0.17
socialscience_aptitude	0.30 *	0.10	0.20	0.24 *	0.02	−0.05	−0.30 **
sports_aptitude	0.01	0.09	0.06	0.07	−0.11	0.44 ***	0.07
philosophy_aptitude	0.15	0.21	0.44 ***	0.34 **	0.00	−0.28 *	−0.34 **
law_aptitude	0.31 *	0.18	0.43 ***	0.38 **	−0.12	−0.23 *	−0.28 *
religion_aptitude	0.24	0.21	0.32 **	0.32 **	−0.17	−0.03	−0.10
general_aptitude	0.37 **	0.39 **	0.57 ***	0.55 ***	0.13	−0.01	−0.27 *
Humanities Index	0.21	0.10	0.28 *	0.24	−0.24 *	−0.08	−0.25 *
Exact Sciences Index	0.03	0.14	−0.10	0.02	0.36 **	0.14	−0.15
Multiple Aptitudes	0.49 ***	0.59 ***	0.55 ***	0.66 ***	0.15	0.24 *	−0.12
art_interest	0.18	0.29 *	0.26 *	0.30 *	−0.02	−0.12	0.02
tech_interest	0.17	0.20	0.04	0.16	0.30 **	0.14	−0.11
math_interest	0.32 **	0.27 *	0.24 *	0.34 **	0.44 ***	0.10	−0.27 *
socialscience_interest	0.21	0.07	0.28 *	0.23	0.08	−0.17	−0.37 **
sports_interest	0.01	0.06	−0.02	0.02	−0.06	0.38 **	0.13
philosophy_interest	0.30 *	0.28 *	0.46 ***	0.43 ***	−0.01	−0.14	−0.36 **
law_interest	0.38 **	0.24	0.46 ***	0.44 ***	−0.07	−0.10	−0.27 *
religion_interest	0.09	0.06	0.27 *	0.18	−0.19	−0.13	−0.15
general_interest	0.39 **	0.38 **	0.50 ***	0.52 ***	0.10	−0.01	−0.30 **

**Table 3:** Correlations of the BIS-11 scales with the documented adult university grades on the Exam, Participation (Partic), Assignments (Asgmt), Oral Presentations (OralPr), Grade Average (GradeAv), and estimated high-school grades in science and math subjects (GrSci) (*N* = 151) and the STQ-77 scales (*N* = 296). Other grade-related variables did not have significant correlations with BIS-11. * *p* < 0.05, ** *p* < 0.01, *** *p* < 0.001.

Scales:	LoP	LoA	MoM	BIS11	Exam	Partic	Asgmt	OralPr	GradeAv
*N*=	153	153	153	153	88	88	53	35	88
Exam	−0.15	−0.20	−0.18	−0.22 *	1	0.36 **	0.24	0.28	0.85 ***
Asgmt	−0.34 **	−0.25	−0.18	−0.36 **	0.24	0.14	1	0.04	0.67 ***
GradeAv	−0.26 *	−0.23 *	−0.26 *	−0.33 **	0.85 ***	0.64 **	0.67 ***	0.49 **	1
GrSci	−0.04	−0.25 **	−0.13	−0.16	0.36 **	0.11	0.10	0.004	0.29 **
ERI	−0.09	−0.57 ***	−0.40 ***	−0.42 ***	0.14	0.19	0.27 *	0.42 *	0.23 *
PL	0.12	−0.18 *	−0.09	−0.04	−0.13	−0.25 *	0.13	−0.06	−0.15
PRO	−0.07	−0.36 ***	−0.39 ***	−0.33 ***	0.28 **	0.30 **	0.29 *	0.44 **	0.39 ***
ERS	0.16	0.08	0.28 **	0.23 **	−0.16	−0.17	−0.16	0.55 **	−0.14
TMS	0.05	−0.08	0.11	0.05	−0.09	−0.16	−0.20	0.34 *	−0.16
EMP	0.22 **	−0.03	0.14	0.17 *	0.08	0.07	0.05	0.13	0.08
ERM	0.07	−0.01	0.00	0.04	−0.01	−0.05	−0.21	0.11	−0.08
TMM	0.03	0.03	0.08	0.06	−0.10	−0.07	−0.26	−0.10	−0.16
SS	0.25 **	0.14	0.33 ***	0.33 ***	−0.08	−0.14	−0.30 *	−0.18	−0.20
SLF	0.19 *	−0.02	0.03	0.11	−0.01	−0.05	−0.21	0.14	−0.08
IMP	0.12	0.28 ***	0.59 ***	0.42 ***	−0.08	−0.12	−0.22	0.04	−0.16
NEU	−0.20 *	0.30 ***	0.21 *	0.08	0.05	−0.04	0.10	−0.27	0.03

**Table 4:** Correlations of the STQ-77 scales with aptitudes (*N* = 80) and interests (*N* = 79). *Note:* STQ-77 scales: ERM/S/I—Motor/Social/Intellectual Endurance; TMM—Motor Tempo; SS—Sensation Seeking; EMP—Empathy; PL—Plasticity; PRO—probabilistic processing; IMP—Impulsivity; NEU—Neuroticism. Zeros before the decimal points are omitted. The Social Tempo and Satisfaction scales did not have significant correlations with aptitudes and interests. * *p* < 0.05, ** *p* < 0.01, *** *p* < 0.001.

Scales:	ERI	PL	PRO	ERS	EMP	ERM	TMM	SS	IMP	NEU
business_aptitude	0.05	0.20	0.08	0.11	−0.25 *	0.37 ***	0.25 *	0.03	0.32 ***	−0.08
art_aptitude	−0.12	0.15	0.01	0.25 *	−0.04	0.06	0.13	0.34 ***	0.06	−0.16
tech_aptitude	0.19	0.01	0.39 ***	0.00	−0.14	0.17	−0.01	−0.07	0.11	−0.06
math_aptitude	0.28 *	−0.11	0.19	0.06	−0.04	0.06	−0.09	−0.13	0.11	−0.08
socialscience_aptitude	0.30 **	0.09	0.20	−0.06	0.19	−0.12	0.03	−0.01	−0.29 **	−0.18
sports_aptitude	−0.05	0.01	−0.15	0.00	0.11	0.51 ***	0.58 ***	0.25 *	0.36 ***	0.16
philosophy_aptitude	0.16	0.16	0.19	0.12	0.16	−0.18	−0.06	0.18	−0.05	−0.28 *
religion_aptitude	0.14	0.20	0.21	0.18	0.07	0.12	0.04	0.12	0.03	−0.22 *
general_aptitude	0.27 *	0.19	0.33 **	0.21	0.05	0.16	0.16	0.16	0.11	−0.28 *
humanities index	0.29 **	0.28 *	0.19	0.11	0.21	−0.09	−0.06	0.20	−0.36	−0.20
multiple aptitudes	0.25 *	0.18	0.32	0.13	0.15	0.26 *	0.20	0.15	0.22	−0.11
business_interest	−0.09	0.12	−0.08	0.02	−0.26 *	0.08	0.06	0.11	0.08	0.02
art_interest	0.06	0.14	0.17	0.27 *	0.19	−0.09	−0.05	0.19	−0.06	−0.18
lifescience_interest	0.05	0.27 *	0.19	0.08	0.16	0.10	0.05	0.00	0.04	−0.21
tech_interest	0.06	0.04	0.29 *	−0.10	−0.13	0.25 *	0.16	−0.09	0.02	−0.06
math_interest	0.34 **	0.11	0.41 ***	−0.03	−0.01	0.19	0.00	−0.18	−0.04	−0.24 *
socialscience_interest	0.35 ***	0.12	0.33 **	0.00	0.24 *	−0.03	−0.18	−0.01	−0.22 *	−0.20
sports_interest	−0.11	−0.07	−0.17	0.01	0.22 *	0.40 ***	0.46 ***	0.10	0.28 *	0.11
philosophy_interest	0.34 **	0.16	0.32 **	0.11	0.27 *	−0.06	−0.11	0.03	−0.11	−0.32 **
law_interest	0.23 *	0.14	0.27 *	0.13	0.03	0.05	−0.04	0.00	−0.22	−0.22
religion_interest	0.12	0.25 *	0.15	0.29	0.15	0.10	0.00	0.06	−0.01	−0.29 **
general_interest	0.31 **	0.28 *	0.43 ***	0.18	0.21	0.22	0.08	0.04	−0.06	−0.36 ***

**Table 5:** The summary of the polymathy profile based on temperament scales of STQ-77, BIS-11, types of aptitudes, and interests. Only significant at *p* < 0.01 positive (+) or negative (−) correlations are shown in this summary.

	Trait	Polymathy	Scales	TPS
	Depth	Breadth	Integration	Total
**STQ-77, temperament profile**				
Intellectual Endurance	**+**	**+**	**+**	**+**
Plasticity		**+**	**+**	**+**
Probabilistic Processing	**+**	**+**	**+**	**+**
Social Endurance			**+**	**+**
Sensation Seeking		**+**	**+**	**+**
Disp. Satisfaction		**+**	**+**	
Neuroticism		−	−	−
**BIS-11** Lack of attention		−		−
**Aptitudes and interests:**				
Philosophy aptitude			**+**	**+**
Law-aptitude			**+**	**+**
Religion-aptitude			**+**	**+**
General/overall aptitudes	**+**	**+**	**+**	**+**
Multiplicity of interests	**+**	**+**	**+**	**+**
Mathematics interests	**+**	**+**		**+**
Law-interests	**+**		**+**	**+**
Philosophy-interests			**+**	**+**
General/overall interests	**+**	**+**	**+**	**+**

## Data Availability

Data is available at https://psonline.ca/Data for Grades-Server.xlsx. (accessed on 1 January 2026).
